# Treatment approaches for non-metastatic small cell bladder cancer: a meta-analysis of reconstructed Kaplan–Meier curves

**DOI:** 10.1016/j.ctro.2025.101032

**Published:** 2025-08-13

**Authors:** Lucas Mose, Priyamvada Maitre, Pascal Eberz, Thomas Zilli, Osama Mohamad, Vedang Murthy, Christian D. Fankhauser, Bernhard Kiss, Beat Roth, Daniel M. Aebersold, Ursula Vogl, Mohamed Shelan

**Affiliations:** a Department of Radiation Oncology, Inselspital, Bern University Hospital, University of Bern, Switzerland; b Department of Radiation Oncology, ACTREC, Tata Memorial Centre, and Homi Bhabha National Institute (HBNI), Mumbai, India; c Department of Radiation Oncology, Oncology Institute of Southern Switzerland, EOC, Bellinzona, Switzerland; d Faculty of Biomedical Sciences, Università Della Svizzera Italiana, Lugano Switzerland; e Faculty of Medicine, University of Geneva, Geneva, Switzerland; f Department of Radiation Oncology, MD Anderson Cancer Center, Houston, TX, USA; g Department of Urology, Luzerner Kantonsspital, Lucerne, Switzerland; h Department of Urology, University Hospital of Bern, University of Bern 3010 Bern, Switzerland; i Oncology Institute of Southern Switzerland (IOSI), Ente Ospedaliero Cantonale (EOC), Bellinzona, Switzerland

**Keywords:** Small cell bladder cancer, Radical cystectomy, Radiotherapy, Chemotherapy, Overall survival

## Abstract

**Introduction:**

Small cell bladder cancer is an aggressive histological subtype and represents one of the most common extra-pulmonary small cell carcinomas. Treatment options include radical cystectomy, chemotherapy, radiotherapy, or combinations of these three. However, the optimal treatment approach remains unknown. This *meta*-analysis assesses the current literature on non-metastatic SCBC and analyzes different treatment approaches.

**Methods:**

A comprehensive search was conducted in four electronic databases (PubMed, Scopus, Web of Science, and Cochrane Library) utilizing the search terms “small or neuroendocrine AND cancer OR Carcinoma OR Tumor OR Malignan* OR oncolog* OR metastati* OR neoplasm AND bladder or urothelial or Urologic” from inception until March 2024. Eligible papers reported treatment and overall survival (OS) of non-metastatic SCBC patients. A *meta*-analysis was conducted comparing treatments with radical cystectomy- and radiotherapy-based approaches in addition to the use of chemotherapy.

**Results:**

In total, 12 articles were included in the present systematic review and *meta*-analysis. Regarding the comparison between radical cystectomy-based treatment and radiotherapy-based treatment, comparable OS was observed between both groups as the reported hazard ratio (HR) was found to be 1.04 (95 % confidence intervals [CI]: 0.90, 1.20, p = 0.6). After including chemotherapy in the analysis, it was observed that using radical cystectomy or radiotherapy with the addition of chemotherapy showed better OS with lower HR (0.53 [95 %CI: 0.39, 0.73], p < 0.0001) compared with radical cystectomy or radiotherapy without chemotherapy. The median OS of radical cystectomy or radiotherapy with chemotherapy was higher than that of radical cystectomy or radiotherapy without chemotherapy, and chemotherapy alone as follows: 30.89 (95 %CI: 23.82, 40.08), 19.67 (95 %CI: 16.26, 23.80), and 19.20 (95 %CI: 16.55, 22.28), respectively.

**Conclusion:**

In this systematic review and *meta*-analysis, no OS difference was observed in patients undergoing radical cystectomy-based or radiotherapy-based treatments. The addition of chemotherapy to local therapy seems to improve OS. However, given the high heterogeneity of the included studies, these results should be interpreted cautiously.

## Highlights

• Small cell bladder cancer is one of the most common extra-pulmonary small cell carcinoma.
• OS is comparable between radical cystectomy- and radiotherapy-based treatment for non-metastatic small cell bladder cancer.
• The addition of chemotherapy to either radical cystectomy or radiotherapy significantly improves OS.

## Introduction

Small cell bladder cancer (SCBC) is a rare and highly aggressive subtype of bladder cancer [1,2]. It represents one of the most common extrapulmonary small cell carcinomas and accounts for approximately 1 % of bladder malignancies [1,2]. Recent data suggest that SCBC may derive from urothelial carcinoma with urothelial-to-neural plasticity [3]. Most frequently, it is diagnosed in men at the age of around 65 years, typically presenting with gross hematuria [1,4]. Given its high metastatic potential, most SCBC cases are diagnosed with nodal involvement and/or distant metastases at the time of presentation [1,4]. Consequently, the overall survival (OS) for patients with SCBC remains poor [1].

Due to the morphological, genetic, and immunohistochemical similarities between SCBC and small cell lung cancer, such as chromogranin, synaptophysin, and CD56 (neural cell adhesion molecule) positivity or lesions in the RB1 (RB transcriptional corepressor 1) and TP53 (tumor protein p53) gene, treatment strategies for SCBC are frequently adapted from small cell lung cancer protocols [1,4,5]. However, recent research indicates distinct mutagenesis patterns between SCBC and small-cell lung cancer [6]. While small cell lung cancer mutations are predominantly tobacco-induced, SCBC mutations primarily arise from APOBEC (apolipoprotein B mRNA editing enzyme)-mediated processes [6]. Additionally, certain mutations, such as TERT (telomerase reverse transcriptase) mutations, are present in SCBC but are less common in small-cell lung cancer [[5], [6], [7]].

Moreover, SCBC may harbor other therapeutically targetable mutations, including PD-L1 (programmed cell death ligand-1), PIK3CA (phosphatidylinositol-4,5-bisphosphate 3-kinase catalytic subunit alpha), DLL3 (delta-like canonical notch ligand 3), and ERBB2 (erb-b2 receptor tyrosine kinase 2) mutations. However, these targets have yet to be validated in clinical practice [6,7]. Thus, for patients with metastatic SCBC, palliative platinum-based chemotherapy remains the standard treatment [1,8]. For non-metastatic SCBC, treatment options include radical cystectomy, chemotherapy, and radiotherapy, either as monotherapy or in combination [4,8]. Consequently, determining the optimal therapeutic approach is challenging due to the absence of prospective trials and the prevalence of limited retrospective studies in the literature.

This study aims to review the current literature on non-metastatic SCBC systematically and to analyze the treatment approaches. Specifically, radical cystectomy-based treatments are compared to radiotherapy-based treatments, and the efficacy of local therapy, encompassing radical cystectomy or radiotherapy, with or without chemotherapy, to chemotherapy alone.

## Methods

Adhering to the Cochrane Handbook of Systematic Reviews of Interventions at each step [9]. Following the Preferred Reporting Items for Systematic Reviews and Meta-Analyses (PRISMA) statement’s guidelines, we conducted this systematic review and *meta*-analysis [[10], [11], [12]]. The protocol of this study was registered at PROSPERO (CRD42024581603).

### Eligibility criteria

The study population included articles reporting follow-up on non-metastatic SCBC patients, with any intervention indicated, including radiotherapy, radical cystectomy, chemotherapy, or a combination of any of them. The included articles were divided into two groups for the pairwise *meta*-analysis (radical cystectomy with or without chemotherapy and radiotherapy with or without chemotherapy). Patients were also divided into three arms: radiotherapy or radical cystectomy without chemotherapy, radiotherapy or radical cystectomy with chemotherapy, and chemotherapy alone. The primary outcome was OS. Study design included randomized controlled trials (RCTs) and observational studies, including cohort and case-control studies. Case reports, reviews, and *meta*-analyses were excluded.

### Information sources and search strategy

We performed a comprehensive search of 4 electronic databases (PubMed, Scopus, Web of Science, and Cochrane Library): Small or neuroendocrine AND cancer OR Carcinoma OR Tumor OR Malignan* OR oncolog* OR metastati* OR neoplasm AND bladder or urothelial or Urologic. Additionally, the references of the included studies were manually searched for potentially eligible studies.

### Selection process

The resulting articles from the literature search were incorporated in EndNote software (ClarivateAnalytics) to remove the duplicates, followed by screening the retrieved references in two steps. The first step was the title and abstract screening to test the eligibility of the inclusion of the studies by their title and abstract, followed by a full-text review of the included articles from the previous step. Each step was conducted by three authors who worked independently, and any disagreements were settled by consensus, and if any of them persisted, a senior author resolved them.

### Data extraction

We extracted the baseline data of the included studies, including the study design, country, sample size, age, gender, and the outcome data related to OS.

### Quality assessment

Two independent researchers conducted the quality assessment and risk of bias assessment, and any disagreements were resolved by consensus or by the senior author. The New Castle Ottawa Scale (NOS) [13], which assigns a star rating to each study between 0 and 9, was used to assess the quality of observational studies [[14], [15], [16]]. Every question has the option to receive one, two, or zero stars, except for the comparison question, which can receive two stars. If a study gets 1 to 3 stars, it is considered low quality; if it gets 4 to 6 stars, it is considered moderate quality; and if it gets 7 to 9 stars, it is considered high quality. Additionally, the risk of bias was assessed with the ROBINS-I tool, which evaluates bias across seven domains. The overall risk assessment was determined based on the domain with the highest risk of bias [17].

### Statistical analysis

The *meta*-analysis of hazard ratio (HR) obtained from Kaplan-Meier survival curves was conducted using RevMan software [18] when at least three studies provided data on the evaluated outcomes. For time-to-event outcomes, including OS, HRs with 95 % confidence intervals (CIs) were calculated. Survival data, available only in Kaplan–Meier curves, were digitized using the Plot Digitizer online tool [19]. A reconstructed *meta*-analysis was performed, and HRs with 95 % CIs were derived using a validated method outlined by Tierney et al. [20,21]. Statistical significance was defined as p < 0.05. Inverse variance method was applied to produce the forest plots using HR and 95 % CIs. A statistical analysis was performed to calculate the pooled median OS times and investigate the heterogeneity across studies. Reported median survival times and their 95 % confidence intervals (CIs) were log-transformed to approximate normality. To estimate the pooled log-transformed medians, a random effects model (RE model) using the DerSimonian and Laird method was applied [22]. These medians were subsequently transformed back to the original scale. Heterogeneity was measured by Tau^2^ which is the term used for the between-study variance. Tau^2^ along with its 95 % CI was estimated using the Q-profile method. Results were accompanied by 95 % CI and forest plots were used to present the individual study estimates and the pooled estimate [23]. All analyses were conducted with the metafor package in R (version 4.4.1) [24].

## Results

### Literature search and screening

The literature search resulted in a total of 10,104 studies from the searched databases. We removed a total of 4993 duplicates and the screening was conducted on the remaining 5111 articles. After title and abstract screening, we included a total of 25 articles, from which 12 articles [8,[25], [26], [27], [28], [29], [30], [31], [32], [33], [34], [35]] were included in the current systematic review and *meta*-analysis after full-text screening. A PRISMA flowchart describing the process and selection of included studies is provided in (Supplementary Fig. 1).

### Quality assessment

The quality assessment of the included cohort studies, using both ROBINS-I and NOS, revealed an overall moderate risk of bias across studies, with confounding and the selection of reported results being the primary sources of concern. The majority of studies demonstrated low risk in domains such as participant selection, intervention measurement, and outcome assessment. Patient selection bias and confounding may have influenced the study’s conclusions by affecting treatment group comparability. Selection may have affected the overall results, potentially skewing survival outcomes. Additionally, differences in chemotherapy use, staging methods, and tumor histology introduce confounding factors that limit definitive treatment comparisons and may have affected the selection as well. On the NOS, study quality ranged from moderate to high, with higher scores reflecting strong methodological rigor in areas like cohort representativeness, exposure ascertainment, and outcome assessment. These findings indicate that while the studies were generally well-designed, some limitations, particularly in confounding and cohort comparability, may affect the interpretation of their results (Table 1 and Supplementary Table 1).

**Table 1:** Quality assessment of the included cohort studies using ROBINS-I.

Domain	Ismaili et al, 2008	Lynch et al, 2012	Schreiber et al, 2013	Kaushik et al, 2015	Eswara et al, 2015	Fischer-Valuck, et al 2017	Akamatsu et al, 2019	Nayeri et al, 2020	Grigg et al, 2020	Chau et al, 2021	Oh, et al, 2021	Yuen Teo et al, 2022
Bias due to confounding	Moderate risk	Moderate risk	Moderate risk	Moderate risk	Moderate risk	Moderate risk	Moderate risk	Moderate risk	Moderate risk	Moderate risk	Moderate risk	Moderate risk
Bias in selection of participants into the study	Low risk	Low risk	Low risk	Low risk	Low risk	Low risk	Low risk	Low risk	Low risk	Low risk	Low risk	Low risk
Bias in measurement of interventions	Low risk	Low risk	Low risk	Low risk	Low risk	Low risk	Low risk	Low risk	Low risk	Low risk	Low risk	Low risk
Bias due to departures from intended interventions	Low risk	Low risk	Low risk	Low risk	Low risk	Low risk	Low risk	Low risk	Low risk	Low risk	Low risk	Low risk
Bias due to missing data	Low risk	Low risk	Low risk	Low risk	Low risk	Low risk	Low risk	Low risk	Low risk	Low risk	Low risk	Low risk
Bias in measurement of outcomes	Low risk	Low risk	Low risk	Low risk	Low risk	Low risk	Low risk	Low risk	Low risk	Low risk	Low risk	Low risk
Bias in selection of the reported result	Moderate risk	Moderate risk	Moderate risk	Moderate risk	Moderate risk	Moderate risk	Moderate risk	Moderate risk	Moderate risk	Moderate risk	Moderate risk	Moderate risk
Overall	Moderate risk	Moderate risk	Moderate risk	Moderate risk	Moderate risk	Moderate risk	Moderate risk	Moderate risk	Moderate risk	Moderate risk	Moderate risk	Moderate risk

### Baseline characteristics

We included 12 cohort studies in the present systematic review with a total sample size of 1814 SCBC patients. Seven of these studies were conducted in the USA, and one each in Iran, the UK, Japan, Columbia, and France. The mean age of patients ranged from 60.5 to 72.5 years old and most of them were males (∼70–100 %). Five articles reported on patients diagnosed with pure SCBC histology only, and seven articles included SCBC with pure and mixed histology. The baseline characteristics of the included studies are summarized in Table 2).

**Table 2:** Baseline characteristics of the included studies.

Study ID	Study design	Sample size	Age, mean (SD)	Male, n (%)	Med.FU	Histology Type (n)	Regional lymph node status (n)	Local therapies	Timing of Chemotherapy	Chemotherapy details (agent, dose and cycles)	Pattern of first recurrences
Yuen Teo et al, 2022	Cohort	124	69.5 (12)	97 (78)	RC-only: 9.1 years; Neoadj. Chemo:7.2 years	Pure SCBC	Node negative: 91 Node positive: 14 Unknown: 19	RT/RC	Adjuvant and Neoadjuvant	platinum-etoposide or cyclophosphamide/doxorubicin/vincristine	Liver, lung, brain, bone, non-regional lymph nodes, Loco-regional recurrence
Schreiber et al 2013	Cohort	297	72.5 (10.8)	231 (77.8)	12 months	Pure SCBC	Node negative: 224 Node positive: 73	RT/RC	NR	NR	NR
Nayeri et al, 2020	Cohort	13	64.92 (10.5)	6 (77)	NR	Pure SCBC (10) Mixed (3)	Node negative: 8 Node positive: 5	RC	NR	NR	NR
Fischer-Valuck et al, 2017	Cohort	693	18–90	541 (78)	18.3 months	Pure SCC	Node negative	RT/RC	Adjuvant and Neoadjuvant	NR	NR
Eswara, et al, 2015	Cohort	28	66 (17.4)	12 (72)	34 months	Pure SCBC (24) Mixed (4)	Node negative: 22 Node positive: 6	RT/RC	NR	4 cycles of cisplatin 50–70 mg/m2 or carboplatin 400 mg/m2 and etoposide 75 mg/m2. Other agents: gemcitabine, ara-C, adriamycin, cyclophosphamide, taxol, methotrexate, vinblastine, melphalan, and estramustine.	Pelvic lymph nodes, paraaortic lymph nodes, bone (left femur and scapula, right iliac crest), bladder, pelvic sidewall, lung, brain, adrenal glands
Chau et al, 2021	Cohort	409	70.8 (10.3)	306 (74.3)	15.4 months	Pure SCBC (189) Mixed (220)	Node negative: 237 Node positive: 172	RT/RC	Neoadjuvant	Carboplatin or cisplatin with etoposide or gemcitabine	NR
Akamatsu et al, 2019	Cohort	12	70.5 (10.75)	11 (91.7)	27.3 months	Purce SCBC (5) Mixed (3) Unknown (4)	Node negative: 10 Node positive: 2	RT	Adjuvant and Neoadjuvant	Carboplatin or cisplatin with etoposide, gemcitabine or irinotecan	Liver, bones, *para*-aortic lymph nodes, lungs, and brain
Lynch et al, 2012	Cohort	67	65.8 (12.7)	55 (82)	NR	Pure SCBC (25) Mixed (42)	Node negative: 59 Node positive: 8	RT/RC	Adjuvant and Neoadjuvant	Utilized agents with the following combinations: IA = ifosfamide, doxorubicin; EP = etoposide, cisplatin; MVAC = methotrexate, vinblastine, doxorubicin, cisplatin; TMP = paclitaxel, methotrexate, cisplatin; CGI = cisplatin, gemcitabine, ifosfamide; EAP = etoposide, doxorubicin, cisplatin; GCtx = gemcitabine, cyclophosphamide; GTA = gemcitabine, doxorubicin, paclitaxel; ECarbo = etoposide, carboplatin; VACtx = vincristine, doxorubicin, cyclophosphamide; 5-FUCarbo = 5-fluorouracil, carboplatin.	NR
Kaushik et al, 2015	Cohort	68	69 (2.5)	68 (85)	8.7 years	Pure SCBC	Node negative: 44 Node positive: 24		Adjuvant	NR	NR
Grigg et al, 2020	Cohort	30	70 (9.6)	24 (80)	39.6 months	Pure SCBC (8) Mixed (22)	Node negative: 28 Node positive: 2	RT/RC	Adjuvant and Neoadjuvant	3–5 cycles of a platinum agent plus etoposide except one who received platinum plus gemcitabine	Lymph nodes and bone metastases
Oh et al, 2021	Cohort	77	70.4 (2.5)	58 (75)	NR	Pure and mixed	Node negative: 54 Node positive: 23	RT/RC	Adjuvant and Neoadjuvant	NR	Local recurrence
Ismaili et al, 2008	Cohort	10	60.5 (8.25)	10 (100)	49 months	Pure and mixed	NR	RC/RT	Adjuvant and Neoadjuvant	Etoposide and cisplatin	Liver and lymph nodes metastases

Abbreviations: SD: standard deviation, RT: radiotherapy, RC: radical cystectomy, SCBC: small cell bladder cancer, NR: nor reported,

### Quantitative synthesis

For the studies without comparison groups, we conducted a quantitative synthesis. Eswara et al. [33] sought to document the clinical and management outcomes of SCBC, noting that patients with T1–2N0M0 disease had longer survival compared to those with advanced disease. Patients with T3–4 or nodal/metastatic illness exhibited enhanced survival rates with treatment. Ismaili et al. [30] documented their experience in managing SCBC and concluded that radical cystectomy combined with neoadjuvant, or adjuvant chemotherapy is a feasible strategy.

### Meta-analysis

#### Kaplan-Meyer analysis

**Fig. 1:**
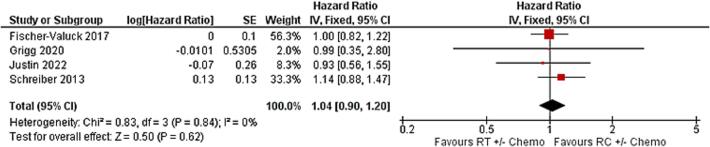
Comparison between radical cystectomy and radiotherapy-based treatments in the overall survival using hazard ratio.

Regarding the comparison between radical cystectomy-based treatment and radiotherapy-based treatment, comparable OS was observed between both groups as the reported HR was found to be 1.04 (95 %CI: 0.90, 1.20, p = 0.6). We did not find evidence that treatment outcomes were different. The heterogeneity was minimal with a p-value of 0.8 (Fig. 1).

**Fig. 2:**
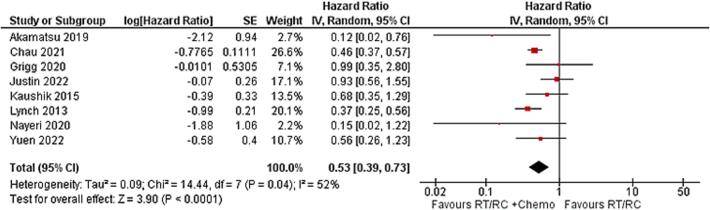
Comparison between radical cystectomy or radiotherapy-based treatments including chemotherapy against radical cystectomy or radiotherapy-based treatments without chemotherapy in the overall survival using hazard ratio.

After including chemotherapy in the analysis, it was observed that using radical cystectomy or radiotherapy with the addition of chemotherapy showed better OS with lower HR (0.53 [95 %CI: 0.39, 0.73], p < 0.0001) compared with radical cystectomy or radiotherapy without chemotherapy. Some heterogeneity was observed as the Tau^2^ = 0.09, and p-value = 0.04 (Fig. 2). Moreover, the use of radical cystectomy or radiotherapy with chemotherapy showed better outcomes compared with chemotherapy alone, with HR = 0.69 (95 %CI: 0.61, 0.69, p < 0.00001). The heterogeneity was minimal as the Tau^2^ = 0, and the p-value was 0.5 (Supplementary Fig. 2). This indicates that the use of local therapies in addition to chemotherapy is associated with the best outcomes for SCBC patients compared with either of the treatments alone.

#### Pooled median overall survival (OS)

**Fig. 3:**
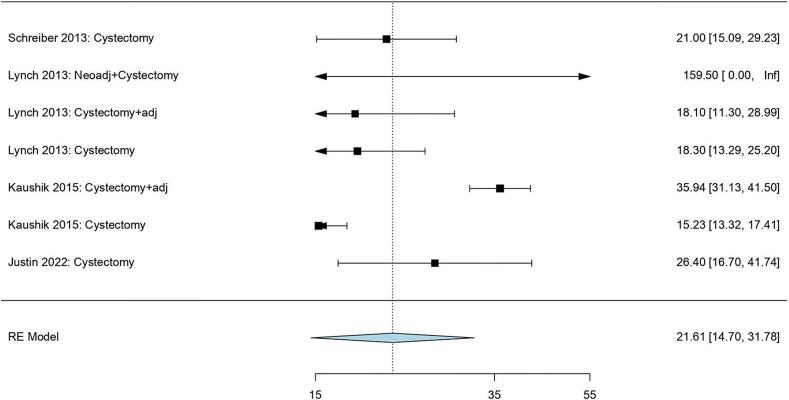
Pooled median overall survival of radical cystectomy-based treatment.

**Fig. 4:**
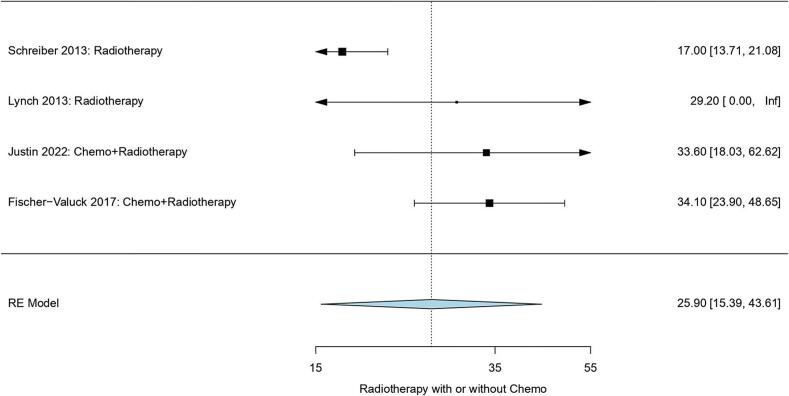
Pooled median overall survival of radiotherapy-based treatment.

The median OS of radical cystectomy-based treatment was reported to be 21.61 months (95 %CI: 14.70, 31.78) while that of radiotherapy-based treatment was observed to be 25.90 months (95 %CI: 15.39, 43.61). Significant heterogeneity was observed in both groups as the parameters of radical cystectomy-based treatment were as follows: Tau^2^ = 0.20, and p-value was < 0.00001, and those of radiotherapy-based treatment were as follows: Tau^2^ = 0.17, and p-value was 0.0005 (Fig. 3, Fig. 4, respectively).

The median OS of radical cystectomy or radiotherapy with chemotherapy was higher than that of radical cystectomy or radiotherapy without chemotherapy, and chemotherapy alone as follows: 30.89 months (95 %CI: 23.82, 40.08), 19.67 months (95 %CI: 16.26, 23.80), and 19.20 months (95 %CI: 16.55, 22.28) (Supplementary Figs. 3, 4 and 5). Minimal heterogeneity was reported for radical cystectomy or radiotherapy with chemotherapy as the parameters were observed as follows: Tau^2^ = 0.035, and p-value = 0.1. Also, minimal heterogeneity was observed in the chemotherapy group with Tau^2^ = 0, and p-value = 0.4. However, significant heterogeneity was observed in the radical cystectomy or radiotherapy without chemotherapy groups, showing Tau^2^ = 0.039, and p-value = 0.009.

### Discussion

To the best of our knowledge, this is the first *meta*-analysis of treatment approaches in patients with non-metastatic SCBC. No statistically significant difference was observed for OS between radical cystectomy and radiotherapy-based treatments. The addition of chemotherapy to local therapy appears to improve OS. These results should be interpreted in the context of diverse patient cohorts treated with varied treatment approaches, with inherent risks of heterogeneity and bias among the studies.

Two of the largest studies − a retrospective analysis of 404 patients with non-metastatic SCBC from the National Cancer Database, and another multicentric study of 165 patients, found no difference in OS between radical cystectomy and radiotherapy-based approaches [32,34]. Some smaller studies did suggest a benefit towards radical cystectomy, yet the risk of selection biases remains a concern, as most studies report a worse prognosis with older age and worse performance status − factors, which also serve as practical criteria for preferring radiotherapy over surgery [[25], [26], [27],^32^]. When these factors are balanced between both groups, OS may not differ, as shown in a small study of 30 patients [33]. These findings are congruent with survival comparisons between radical cystectomy and radiotherapy in urothelial muscle-invasive bladder cancer. In the largest multicentric study of patients with muscle-invasive bladder cancer who would have been medically eligible for both treatment approaches, no difference was observed in oncological outcomes between radical cystectomy and radiotherapy [36]. The lack of radiotherapy details such as treatment intent and prescribed dose of earlier treatments are known limitations of NCDB or SEER databases, making it difficult to draw meaningful comparisons. Additionally, surgically staged node-negative disease may differ in prognosis from clinical staging-based radiotherapy cohorts. Yet, our *meta*-analysis suggests that either of the two treatment approaches may be suitable for patients with non-metastatic SCBC, without compromising survival.

Treatment-related morbidity differs between radical cystectomy and radiotherapy, which was not systematically reported in the included studies [37]. Nonetheless, the radiotherapy-based approach preserves native bladder, with up to 85 % bladder preservation rate reported after 2 years [32]. Additionally, even in the event of a local recurrence, bladder function may be preserved for 7–54 months, which contributes to the patient’s quality of life [35]. Most of the recurrences are predominantly nodal or distant, indicating the need for intensified systemic therapy [30,31,33,35,38].

Systemic therapy regimens used in the included studies were inconsistent in sequencing local treatment or lacked adequate reporting [8,25,27]. The current AUA/ASCO/ASTRO/SUO consensus, as well as the National Comprehensive Cancer Network® (NCCN®) guidelines, recommend neoadjuvant platinum-based systemic therapy with radiotherapy or radical cystectomy for localized SCBC while acknowledging the lack of an established schedule [39,40]. Some of the included studies suggest that neoadjuvant chemotherapy (NACT) may be more favorable than adjuvant chemotherapy [26,29,41,42]. Most studies report the use of platinum-based regimens, consistent with current SCLC treatment guidelines [8,25,26,28,30,31,[33], [34], [35],^39^,^40^]. Response to NACT has been observed to be a prognostic factor for OS, and downstaging to ≤pT1N0 was seen in 48–62 % of patients undergoing neoadjuvant chemotherapy before undergoing local treatment [8,26]. However, the possibility of selection bias during decision-making for NACT versus primary local treatment cannot be ruled out. In our analysis, combined local and systemic treatment yielded the highest OS as compared to monotherapy. Extrapolating from the evidence for NACT in urothelial muscle-invasive bladder cancer, it would appear reasonable to use the response to chemotherapy as a form of selection for planning local treatment [43].

Treatment approaches for localized SCBC are often a combination of strategies extrapolated from urothelial muscle-invasive bladder cancer and small-cell lung cancer, depending on the proportion of small-cell variants in the pathology specimen. Our analysis included 4 studies with pure SCBC, while the others reported on combined pure and mixed cohorts without separating their oncological outcomes. Thus, it is not possible to determine the prognostic effect of the small cell component. In general, our results are in agreement with the available evidence in urothelial muscle-invasive bladder cancer. More recent multicentric data suggests that clinical outcomes are not necessarily worse in the presence of a variant histology for patients with bladder cancer treated with radical radiotherapy [44]. The present *meta*-analysis confirms the efficacy of a combined local and systemic treatment approach, with similar outcomes between surgical and radiotherapy-based approaches.

Despite being the first *meta*-analysis and representing the largest patient cohort of non-metastatic SCBC patients, this study has several limitations. First, all the included studies are retrospective, which may introduce significant selection bias between the treatment groups, as patients in the radical cystectomy group might be in better general condition than those in the radiotherapy group, or vice versa, with patients receiving chemotherapy potentially being healthier than those not receiving chemotherapy [26,27,29]. This is reflected in the ROBINS I quality assessment, in which at least moderate risk has been displayed in all included studies. Second, patient-related data, including the inclusion of patients with pelvic lymph nodal metastases, and treatment details, such as radiation dose, irradiated volume, extent of pelvic lymph node dissection, as well as the dose and timing of systemic treatment, are inconsistently reported and vary between cohorts [25,26,41,42]. Therefore comparisons between the reported groups have to be interpreted cautiously. Third, sample sizes vary between cohorts and include patients diagnosed over a larger period, which may also impact treatment decisions and consequently oncological outcomes [4,8].

Although there are ongoing prospective trials involving SCBC patients, such as NCT05199272 and NCT05760053, investigating the safety or efficacy of systemic agents, these studies also include patients with various other entities in locally advanced or metastatic settings. Since current treatment approaches are predominantly based on retrospective data, those trials might help in shaping the optimal treatment approach for SCBC. Yet, several targetable lesions have been identified within the biological profile of SCBC, highlighting the potential for future research to include transcriptomic profiling of tumor tissue and the application of targeted systemic therapies [[5], [6], [7]]. Given that PD-L1 inhibitors are already introduced in the treatment of bladder cancer, they may hold promise for SCBC, in which PD-L1 expression has been observed [7,45]. Moreover, an in-vivo patient-derived xenograft model demonstrated that DLL-targeted therapy was more effective than traditional chemotherapy, further supporting the role of targeted therapies in SCBC management [7]. However, the rarity of this disease poses a challenge for prospective studies, underscoring the importance of multicenter collaborations to advance research in this area.

## Conclusion

In this *meta*-analysis, no significant OS difference was observed between patients undergoing radical cystectomy-based or radiotherapy-based treatments. The addition of chemotherapy to local therapy seems to improve OS. However, the evidence is limited by the small number of studies with low patient numbers, across study heterogeneity, and the possibility of publication bias**.**

## Limitations

The findings of this *meta*-analysis are subject to considerable heterogeneity, which significantly impacts the validity of pooled estimates. A major source of heterogeneity arises from the comparison of radical cystectomy and radiotherapy without stratifying for chemotherapy administration, leading to substantial variability in overall survival (OS) within treatment arms. Additional contributing factors include differences in chemotherapy regimens, surgical techniques, radiotherapy protocols, and baseline patient characteristics such as tumor stage and histology. Confounding biases may have influenced treatment selection and OS outcomes, further complicating direct comparisons. The ROBINS-I assessment underscores these concerns, as all included cohort studies exhibited a moderate risk of bias, primarily due to confounding and selective reporting. Unmeasured variables may have distorted the observed associations, while selective outcome reporting raises concerns regarding the reliability of certain findings. Given the heterogeneous nature of the data, the pooled estimates should not be interpreted as definitive comparative effectiveness measures but rather as indicative trends that warrant further investigation. Future studies should employ more standardized methodologies to enhance comparability, including uniform reporting of patient characteristics and treatment protocols. However, the rarity of the disease presents a challenge in achieving such standardization. While *meta*-analysis provides valuable insights, the considerable heterogeneity identified in this study underscores the need for cautious interpretation, emphasizing the exploratory rather than confirmatory nature of these findings.

## Author contributions

• Lucas Mose and Priyamvada Maitre authored the introduction, results, conclusion, and abstract, and coordinated manuscript formatting, submission, and revisions.

• Pascal Eberz and Lucas Mose performed the systematic literature search, screened and selected studies, and extracted data. They contributed to the methods section, focusing on study selection criteria, data extraction, and the literature review.

• Lucas Mose and Priyamvada Maitre conducted the meta-analysis, including statistical analyses and interpretation of Kaplan–Meier survival curves. They prepared the statistical methodology section, generated forest plots, and contributed to the methods section.

• Pascal Eberz and Priyamvada Maitre conducted the quality assessment and risk-of-bias analysis for the included studies, using the ROBINS-I tool and the Newcastle–Ottawa Scale. They contributed to the discussion on study limitations and the quality of evidence.

• Osama Mohamad, Vedang Murthy, Christian D. Fankhauser, and Bernhard Kiss provided clinical expertise on small cell bladder cancer. They reviewed and revised the clinical content, particularly in the introduction, treatment sections, and discussion of clinical outcomes.

• Ursula Vogl, Thomas Zilli, Beat Roth and Daniel M. Aebersold contributed to the interpretation of results and the discussion of the implications of the findings.

• Mohamed Shelan led the study design, conducted the final revisions, and reviewed the manuscript to ensure overall consistency, quality, and adherence to the intended content, structure, and style.

## Declaration of competing interest

The authors declare that they have no known competing financial interests or personal relationships that could have appeared to influence the work reported in this paper.
