# Supplementary material for: Treatment approaches for non-metastatic small cell bladder cancer: a meta-analysis of reconstructed Kaplan–Meier curves
Source: Clin Transl Radiat Oncol. 2025 Aug 13;55:101032. doi: 10.1016/j.ctro.2025.101032 (PMC12392672; doi:10.1016/j.ctro.2025.101032)
Supplement: Supplementary Data 1 [file mmc1.docx]

Supplementary Figure 1: PRISMA (Preferred Reporting Items for Systematic Reviews and Meta-Analyses) flowchart summarizing the screening and selection processes


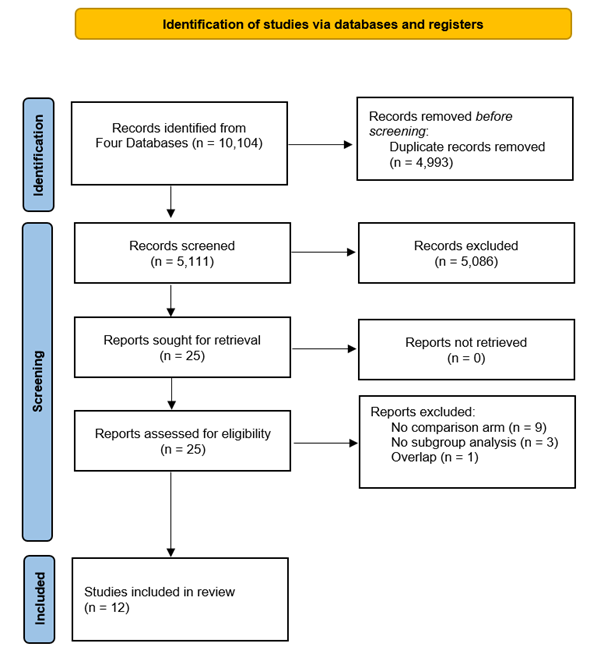


Supplementary Figure 2: Comparison between radical cystectomy or radiotherapy-based treatments including chemotherapy against chemotherapy alone in the overall survival using hazard ratio


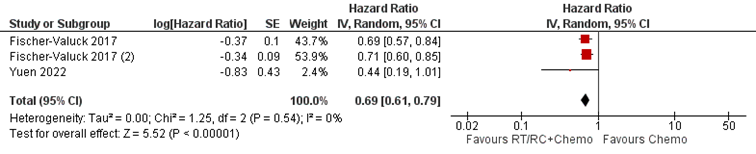


Supplementary Figure 3: Pooled median overall survival of radical cystectomy or radiotherapy-based treatment without chemotherapy


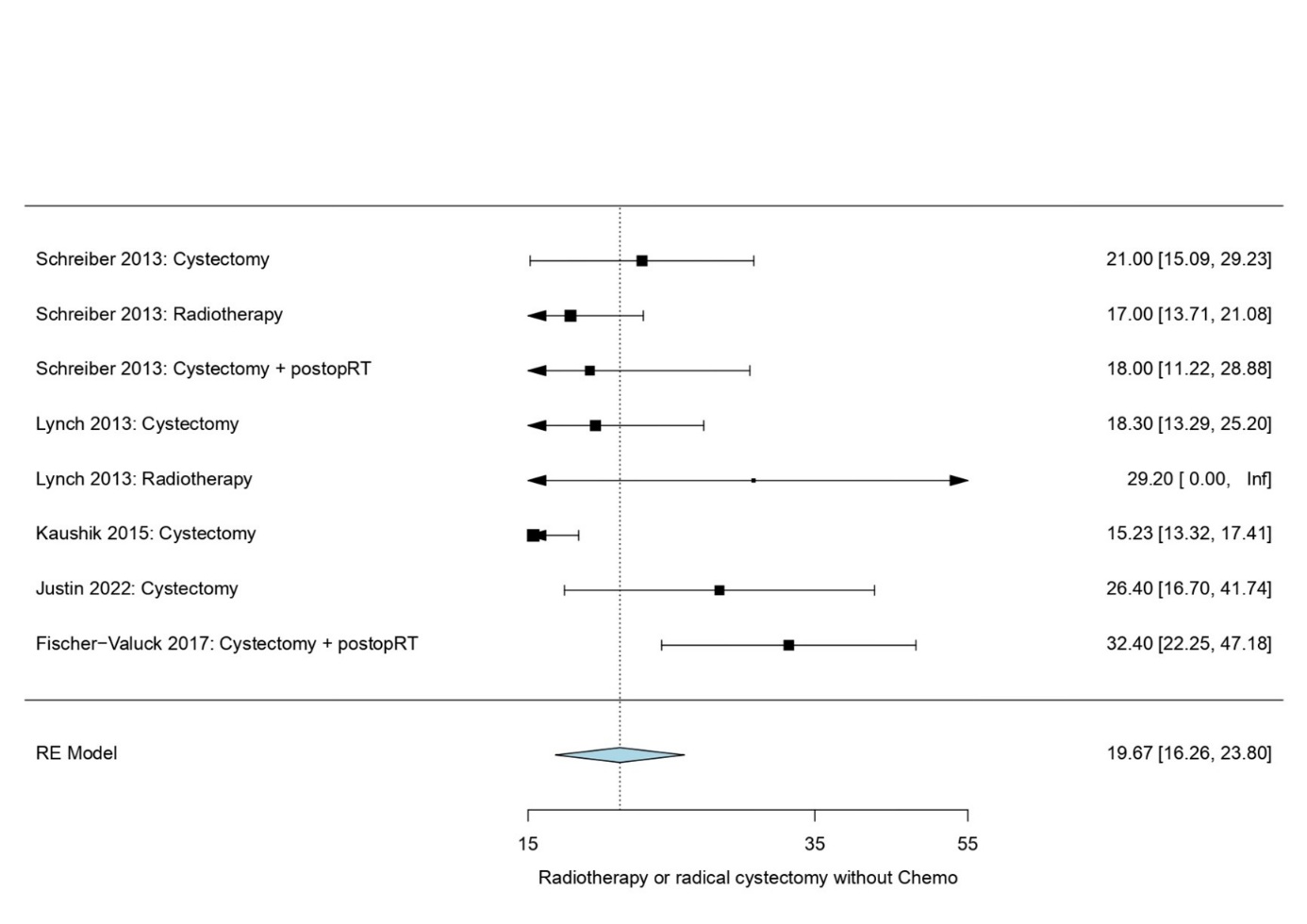


Supplementary Figure 4: Pooled median overall survival of chemotherapy alone


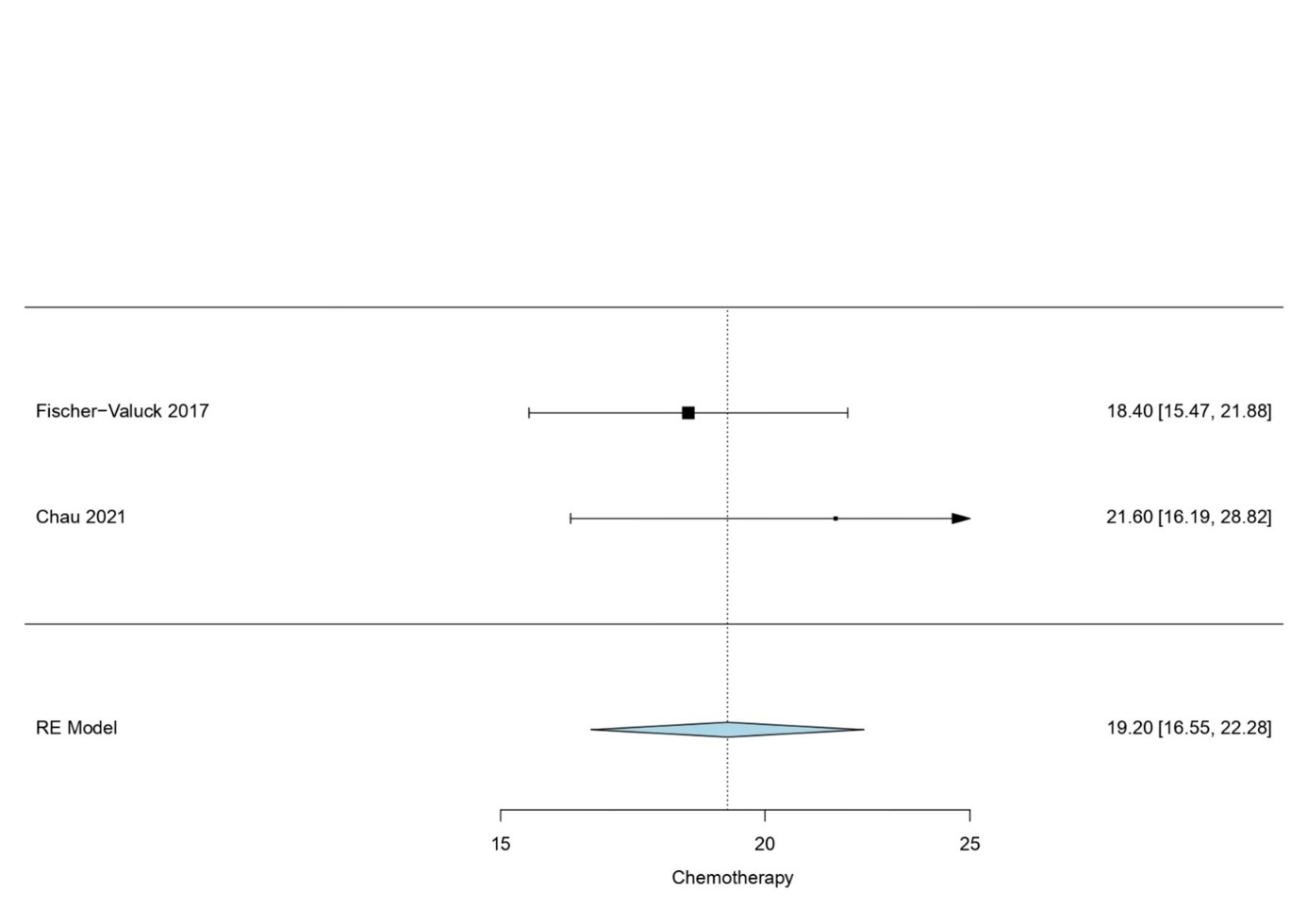


Supplementary Figure 5: Pooles median overall survival of radical cystectomy or radiotherapy-based treatment with chemotherapy


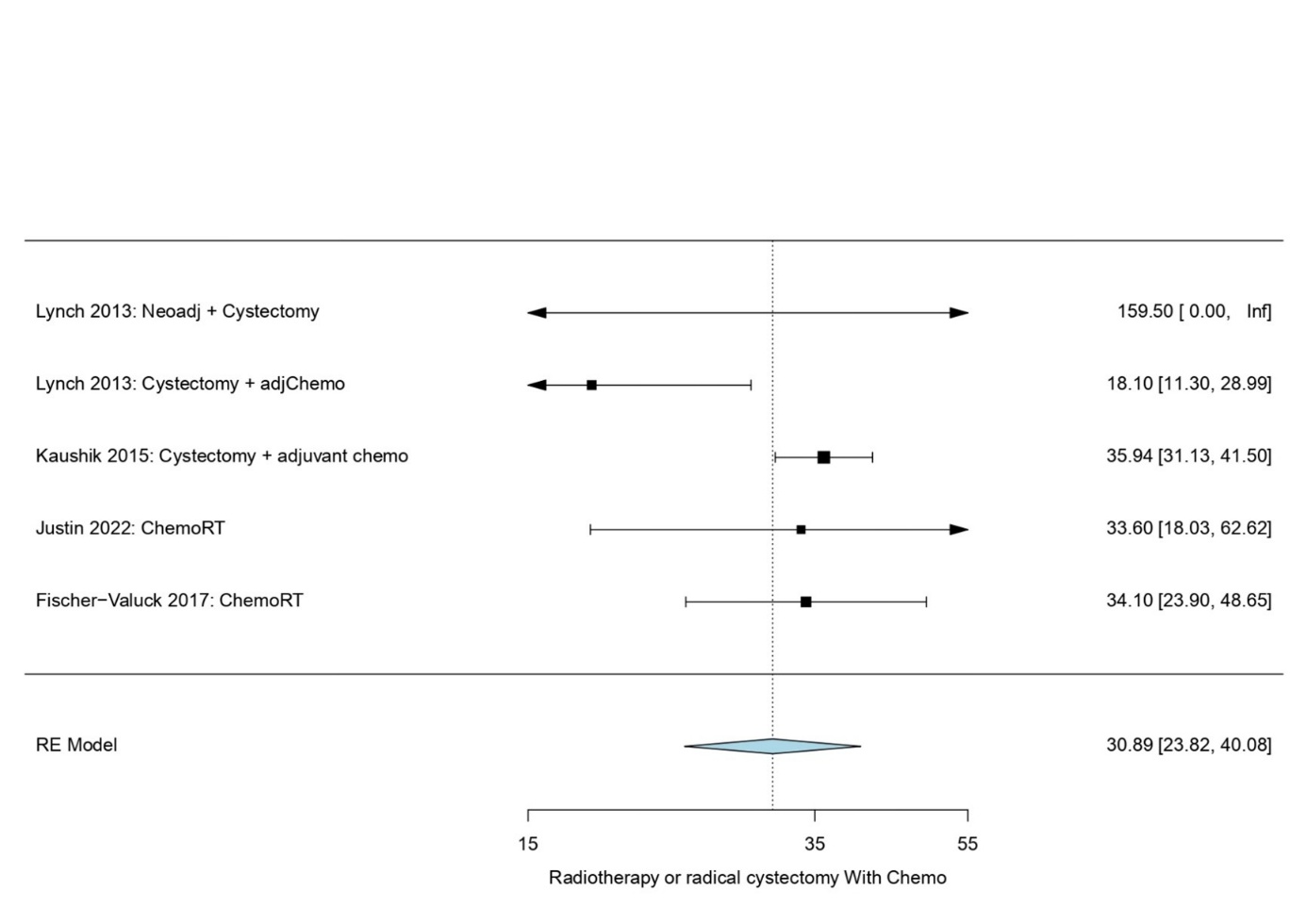


Supplementary Table 1: Quality assessment of the included cohort studies using New Castle Ottawa Scale

| Study name | Representativeness of the exposed cohort (★) | Selection of the non exposed cohort (★) | Ascertainment of exposure (★) | Demonstration that outcome of interest was not present at start of study (★) | Comparability of cohorts on the basis of the design or analysis (max★★) | Assessment of outcome (★) | Was follow-up long enough for outcomes to occur? (★) | Adequacy of follow up of cohorts (★) | Quality level |
| --- | --- | --- | --- | --- | --- | --- | --- | --- | --- |
| Yuen Teo 2022 [12] | ★ | ★ | - | ★ | ★ | ★ | ★ | ★ | High (7) |
| Schreiber 2013 [3] | ★ | ★ | - | - | ★ | ★ | ★ | ★ | Moderate (6) |
| Nayeri 2020 [8] | ★ | ★ | ★ | ★ | ★ | ★ | ★ | ★ | High (8) |
| Fischer-Valuck 2017 [6] | ★ | ★ | ★ | - | ★ | - | ★ | ★ | Moderate (6) |
| Eswara 2015 [5] | ★ | ★ | ★ | - | ★★ | ★ | ★ | ★ | High (8) |
| Chau 2021 [10] | ★ | ★ | ★ | ★ | ★★ | ★ | ★ | ★ | High (9) |
| Akamatsu 2019 [7] | ★ | ★ | - | ★ | ★ | ★ | ★ | ★ | High (7) |
| Lynch 2012 [2] | ★ | ★ | - | - | ★ | ★ | ★ | ★ | Moderate (6) |
| Kaushik 2015 [4] | ★ | ★ | - | - | ★ | ★ | ★ | ★ | Moderate (6) |
| Grigg 2020 [9] | ★ | ★ | ★ | ★ | ★★ | ★ | ★ | ★ | High (9) |
| Oh 2021 [11] | ★ | ★ | ★ | ★ | ★ | ★ | ★ | ★ | High (8) |
| Ismaili 2008 [1] | ★ | ★ | ★ | - | ★★ | ★ | ★ | ★ | High (8) |

References:

1. Ismaili, N., et al., *Small cell cancer of the bladder: The Leon-Berard cancer centre experience.* Indian J Urol, 2008. **24**(4): p. 494-7.

2. Lynch, S.P., et al., *Neoadjuvant chemotherapy in small cell urothelial cancer improves pathologic downstaging and long-term outcomes: results from a retrospective study at the MD Anderson Cancer Center.* Eur Urol, 2013. **64**(2): p. 307-13.

3. Schreiber, D., et al., *Characterization and outcomes of small cell carcinoma of the bladder using the surveillance, epidemiology, and end results database.* Am J Clin Oncol, 2013. **36**(2): p. 126-31.

4. Kaushik, D., et al., *Long-term results of radical cystectomy and role of adjuvant chemotherapy for small cell carcinoma of the bladder.* Int J Urol, 2015. **22**(6): p. 549-54.

5. Eswara, J.R., et al., *Long-term outcomes of organ preservation in patients with small cell carcinoma of the bladder.* Urol Int, 2015. **94**(4): p. 401-5.

6. Fischer-Valuck, B.W., et al., *Treatment Patterns and Survival Outcomes for Patients with Small Cell Carcinoma of the Bladder.* Eur Urol Focus, 2018. **4**(6): p. 900-906.

7. Akamatsu, H., et al., *Organ-preserving approach via radiotherapy for small cell carcinoma of the bladder: an analysis based on the Japanese Radiation Oncology Study Group (JROSG) survey.* J Radiat Res, 2019. **60**(4): p. 509-516.

8. Kaffash Nayeri, R., et al., *Small Cell Carcinoma of Bladder; Still A Diagnostic and Therapeutic Challenge: Seven Years of Experience and Follow-up in A Referral Center.* Urol J, 2020. **17**(4): p. 363-369.

9. Grigg, C.M., et al. *Limited Stage Small Cell Bladder Cancer: Outcomes of a Contemporary Cohort*. 2020.

10. Chau, C., et al., *Treatment Outcomes for Small Cell Carcinoma of the Bladder: Results From a UK Patient Retrospective Cohort Study.* Int J Radiat Oncol Biol Phys, 2021. **110**(4): p. 1143-1150.

11. Oh, J., et al., *Small cell carcinoma of the bladder: A population-based analysis of long-term outcomes after radical cystectomy and bladder conservation with chemoradiotherapy.* Can Urol Assoc J, 2022. **16**(2): p. 55-62.

12. Teo, M.Y., et al., *Long-term Outcomes of Local and Metastatic Small Cell Carcinoma of the Urinary Bladder and Genomic Analysis of Patients Treated With Neoadjuvant Chemotherapy.* Clin Genitourin Cancer, 2022. **20**(5): p. 431-441.
